# Incorporating pharmaceutical supply management modules in the pre-service curriculum of the BPharm program, of the University of Namibia, School of Pharmacy

**DOI:** 10.1186/2052-3211-7-S1-P12

**Published:** 2014-12-17

**Authors:** Greatjoy Njabulo Mazibuko, Evans Sagwa, Harriet Rachel Kagoya, Dan Kibuule, Timothy Rennie, Tukai Mavere, Reem Ghoneim, David Mabirizi, Ester Naikaku, Qamar Niaz, Jennie Lates

**Affiliations:** 1 Management Sciences for Health (MSH)/Systems for Improved Access to Pharmaceuticals and Services (SIAPS), Windhoek, Namibia; 2 University of Namibia School of Pharmacy, Windhoek, Namibia; 3 MSH/SIAPS, Arlington, USA; 4 Ministry of Health and Social Services, Windhoek, Namibia

## Background

Namibia faces a chronic shortage of pharmaceutical personnel. The high burden of HIV and AIDS, coupled with increased numbers of patients needing antiretroviral (ARV) services, has further exacerbated this shortage. Skills related to PSM are essential for ensuring continuous availability of essential medicines for public health programs, including HIV and AIDS and TB. Pre-service education allows students to develop their competencies in supply chain, reducing the need for future investments in expensive in-service training.

## Method

The USAID-funded SIAPS Program facilitated open discussions with University of Namibia lecturers and ministry of health staff to identify PSM components critical for management of medicines at health facilities and include them in the BPharm curriculum. Findings from the discussions, coupled with SIAPS’ prior experience in developing PSM modules for pre-service training in Vietnam, allowed the development of the PSM components, a course outline, method of delivery, and schedule for teaching theory and administering practicals.

## Results

Teaching materials were developed covering 10 procurement and supply chain management (PSM) topics. For each topic, learning objectives, pedagogical techniques, and content summaries were developed. Draft materials for these modules were shared with key stakeholders and workshops conducted to discuss feedback and validate appropriateness for inclusion in the curriculum. The workshops were attended by 15 stakeholders representing University of Namibia, Ministry of Health and SIAPS. As a result, the lecturer’s guide and student materials will be finalized and distributed in July 2014. SIAPS will then collect feedback from lecturers and students and make required improvements.

## Discussion

Routine supervisory support visits to health facilities revealed gaps in Namibia’s public sector supply chain system. These gaps have been largely attributed to lack of competency in SCM, resulting in stock-outs, especially of paediatric ARV formulations. SIAPS has worked collaboratively with stakeholders to enhance pre-service training capacity in PSM. This will ensure that graduates are exposed to PSM techniques necessary to avoid supply chain problems, thus avoiding stock-outs. Cross-linkages between PSM and rational medicine use themes have also been established ensuring that available products are appropriately used.

## Lessons learned

The curriculum has been designed to address the SCM gap in Namibia. Pre-service curriculum development is a sustainable approach worth the investment because it will reduce the future need of more costly in-service trainings. Starting in 2015, pharmacy students will graduate from the University of Namibia equipped with skills and knowledge in PSM.

